# Defective cholesterol metabolism in amyotrophic lateral sclerosis[S]

**DOI:** 10.1194/jlr.P071639

**Published:** 2016-12-29

**Authors:** Jonas Abdel-Khalik, Eylan Yutuc, Peter J. Crick, Jan-Åke Gustafsson, Margaret Warner, Gustavo Roman, Kevin Talbot, Elizabeth Gray, William J. Griffiths, Martin R. Turner, Yuqin Wang

**Affiliations:** Swansea University Medical School,* Swansea, United Kingdom; Department of Biology and Biochemistry,† Center for Nuclear Receptors and Cell Signaling, University of Houston, Houston, TX; Methodist Neurological Institute,§ Methodist Hospital, Houston, TX; Nuffield Department of Clinical Neurosciences,** University of Oxford, John Radcliffe Hospital, Oxford, United Kingdom

**Keywords:** oxysterols, mass spectrometry, cytochrome P450, nuclear receptors/LXR, brain lipids, bile acids and salts/biosynthesis, cholestenoic acids, neurodeneneration.

## Abstract

As neurons die, cholesterol is released in the central nervous system (CNS); hence, this sterol and its metabolites may represent a biomarker of neurodegeneration, including in amyotrophic lateral sclerosis (ALS), in which altered cholesterol levels have been linked to prognosis. More than 40 different sterols were quantified in serum and cerebrospinal fluid (CSF) from ALS patients and healthy controls. In CSF, the concentration of cholesterol was found to be elevated in ALS samples. When CSF metabolite levels were normalized to cholesterol, the cholesterol metabolite 3β,7α-dihydroxycholest-5-en-26-oic acid, along with its precursor 3β-hydroxycholest-5-en-26-oic acid and product 7α-hydroxy-3-oxocholest-4-en-26-oic acid, were reduced in concentration, whereas metabolites known to be imported from the circulation into the CNS were not found to differ in concentration between groups. Analysis of serum revealed that (25R)26-hydroxycholesterol, the immediate precursor of 3β-hydroxycholest-5-en-26-oic acid, was reduced in concentration in ALS patients compared with controls. We conclude that the acidic branch of bile acid biosynthesis, known to be operative in-part in the brain, is defective in ALS, leading to a failure of the CNS to remove excess cholesterol, which may be toxic to neuronal cells, compounded by a reduction in neuroprotective 3β,7α-dihydroxycholest-5-en-26-oic acid.

Amyotrophic lateral sclerosis (ALS) is a heterogeneous, progressive, and fatal neurodegenerative disease characterized by variable loss of upper and lower motor neurons (1). Biomarkers that are sensitive to the progression of disease have the potential to shorten therapeutic trials and provide new drug targets. Study of the metabolome offers the potential to identify disease-specific patterns for ALS, possibly providing such biomarkers and new insights into the deranged biochemical pathways associated with ALS. Metabolomic studies in blood and cerebrospinal fluid (CSF) have been performed utilizing proton nuclear magnetic resonance spectrometry (2–4), GC/MS, and LC/MS (5, 6). Significant differences between patients and controls have been observed, consistent with a range of pathogenic mechanisms that have been described in ALS, including mitochondrial dysfunction, oxidative stress, excitotoxicity, neuroinflammation, and hypermetabolism (7).

Lipids, distinct from other components of the metabolome (8), are nonpolar or amphipathic in nature and require separate analysis from more water-soluble metabolites. Cholesterol, both the nonesterified molecule and its esters with fatty acids, represents a major component of the total lipid content of cells in vertebrates. On a number of fronts, cholesterol and its metabolites represent potential biomarkers for ALS. High plasma levels of cholesterol have been suggested to be neuroprotective for ALS and to be associated with an increased survival time (9–11), but other data suggest that accumulation of cholesterol esters and ceramides mediate oxidative stress in motor neurons in ALS (12), while the gene cytochrome P450 27A1 (*CYP27A1*), encoding cholesterol (25R)26-hydroxylase (also known as sterol 27-hydroxylase), the first enzyme in the extrahepatic part of the bile acid biosynthesis pathway, was recently identified as a susceptibility gene for sporadic ALS (13). In addition, statins, the cholesterol-lowering drugs that inhibit HMG-CoA reductase, have been suggested to accelerate functional decline in ALS patients (14).

ALS is a neurodegenerative disease and as neurons die, cholesterol is released from cells. Cholesterol and its metabolites in the CNS are predominantly in a nonesterified form (15), and it the nonesterified molecules that are responsible for regulating cholesterol homeostasis (16, 17) and are the ligands to nuclear receptors (18, 19) and ligands to G-protein-coupled receptors (20, 21). We aimed to characterize and quantify nonesterified cholesterol and the widest range of nonesterified metabolites in CSF and serum from ALS patients compared with a group of healthy controls (see **Table 1** for molecules analyzed). An LC/MS approach was adopted to exploit charge-tagging to ensure maximum sensitivity and to gain structural information allowing identification of unexpected sterols (supplemental Fig. S1); a hydrolysis step was not included in the sample preparation protocol (22, 23).

**TABLE 1.** Sterols, oxysterols, cholestenoic, and cholenoic acids analyzed in serum and CSF

Systematic Name (trivial name)	Abbreviation	Lipid Maps ID	Authentic Standard
3β-Hydroxycholestatriene*^a^*		NA	No
Cholesta-5,7-dien-3β-ol (7-dehydrocholesterol) + cholesta-5,8-dien-3β-ol*^b^*	7-DHC + 8-DHC	LMST01010069 /NA	Yes
Cholesta-5,24-dien-3β-ol (desmosterol)	24-DHC	LMST01010016	Yes
Cholest-5-en-3β-ol (cholesterol)	C	LMST01010001	Yes
Cholesta-5,8-diene-3β,24(or 25)-diol	24(or 25)H-8DHC	NA	No
Cholesta-5,8-diene-3β,26-diol	26H-8-DHC	NA	No
3β-Hydroxycholest-5-en-7,8-epoxide	7,8-EC	NA	Yes
9,10-Secocholesta-5Z,7E,10-triene-3S,25-diol (25-Hydroxyvitamin D_3_)	25-D_3_	LMST03020246	Yes
9,10-Secocholesta-5Z,7E,10-triene-3S,24,25-triol (24,25-Dihydroxyvitamin D_3_)	24,25-D_3_	LMST03020273 or LMST03020274	Yes
Cholest-4(or 5)-ene-3β,6β-diol (6β-hydroxycholesterol)*^c^*	6β-HC	NA	Yes
Cholest-5-ene-3β,7α-diol (7α-hydroxycholesterol)	7α-HC	LMST01010013	Yes
7α-Hydroxycholest-4-en-3-one	7α-HCO	LMST01010271	Yes
Cholest-5-ene-3β,7β-diol (7β-hydroxycholesterol)	7β-HC	LMST01010047	Yes
3β-Hydroxycholest-5-en-7-one (7-oxocholesterol)	7O-C	LMST01010049	Yes
3β-Hydroxycholest-5-en-24-one (24-oxocholesterol)	24O-C	LMST01010133	Yes
Cholest-5-ene-3β,24S-diol (24S-hydroxycholesterol)	24S-HC	LMST01010019	Yes
Cholest-5-ene-3β,25-diol (25-hydroxycholesterol)	25-HC	LMST01010018	Yes
Cholest-5-ene-3β,(25R)26-diol (27-hydroxycholesterol)	26-HC	LMST01010057	Yes
Cholest-5-ene-3β,12α-diol (12α-hydroxycholesterol)*^d^*	12α-HC	NA	No
Cholest-5-ene-3β,7α,12α-triol (7α,12α-dihydroxycholesterol)*^d^*	7α,12α-diHC	LMST04030165	No
7α,12α-Dihydroxycholest-4-en-3-one*^d^*	7α,12α-diHCO	LMST04030114	No
Cholest-5-ene-3β,7α,25-triol (7α,25-dihydroxycholesterol)	7α,25-diHC	LMST04030166	Yes
7α,25-Dihydroxycholest-4-en-3-one	7α,25-diHCO	LMST04030107	Yes
Cholest-5-ene-3β,7α,26-triol (7α,26-dihydroxycholesterol)	7α,26-diHC	LMST04030081	Yes
7α,26-Dihydroxycholest-4-en-3-one	7α,26-diHCO	LMST04030157	Yes
3β,22-Dihydroxycholest-5-en-24-one*^d^*	22-HC-24-O	NA	No
3β,5α-Dihydroxycholest-7-en-6-one (dihydrocholestenone)		LMST01010260	Yes
Cholesta-5-en-3β,24,25-triol (24,25-dihydroxycholesterol)	24,25-diHC	NA	Yes
3β-Hydroxycholest-5-en-26-oic acid	3β-HCA	LMST04030072	Yes
3-Oxocholest-4-en-26-oic acid	3O-CA	NA	Yes
3β-Hydroxycholesta-5,7-dien-26-oic acid*^e^*	3βH-7-DHCA	NA	Yes
3-Oxocholesta-4,6-dien-26-oic acid*^f^*	3O-6-DHCA	NA	Yes
3β,7α-Dihydroxycholest-5-en-26-oic acid*^e^*	3β,7α-diHCA	LMST04030148	Yes
7α-Hydroxy-3-oxocholest-4-en-26-oic acid*^f^*	7αH,3O-CA	LMST04030149	Yes
Total 3β,7α-Dihydroxycholest-5-en-26-oic acid*^g^*	3β,7α-diHCA	LMST04030148	Yes
Total 7α-Hydroxy-3-oxocholest-4-en-26-oic acid*^h^*	7αH,3O-CA	LMST04030149	Yes
3β,7β-Dihydroxycholest-5-en-26-oic acid	3β,7β-diHCA	NA	Yes
3β-Hydroxy-7-oxocholest-5-en-26-oic acid	3βH,7O-CA	LMST04030215	Yes
3β,22,25-Trihydroxycholest-5-en-24-one*^d^*	22,25-diHC-24-O	NA	No
7α,x,y-Trihydroxycholest-4-en-3-one*^d^*	7α,x,y-triHCO	NA	No
7α,24(or26),25-Trihydroxycholest-4-en-3-one*^d^*	7α,24(or 26),25-triHCO	NA	No
3β,7α,24-Trihydroxycholest-5-en-26-oic acid	3β,7α,24-triHCA	NA	Yes
7α,24-Dihydroxy-3-oxocholest-4-en-26-oic acid	7α,24-diH,3O-CA	NA	Yes
3β,7β,24-Trihydroxycholest-5-en-26-oic aci*^i^*	3β,7β,24-triHCA	NA	No
7β,24-Dihydroxy-3-oxocholest-4-en-26-oic aci*^i^*	7β,24-diH,3O-CA	NA	No
3β,7α,25-Trihydroxycholest-5-en-26-oic acid	3β,7α,25-triHCA	NA	Yes
7α,25-Dihydroxy-3-oxocholest-4-en-26-oic acid	7α,25-diH,3O-CA	NA	Yes
3β,7β,25-Trihydroxycholest-5-en-26-oic aci*^i^*	3β,7β,25-triHCA	NA	No
7β,25-Dihydroxy-3-oxocholest-4-en-26-oic acid*^i^*	7β,25-diH,3O-CA	NA	No
7α,x-Dihydroxy-3-oxocholest-4-en-26-oic acid*^d^*	7α,x-diH,3O-CA	NA	No
3β,7α,12α-Trihydroxycholest-5-en-26-oic acid*^d^*	3β,7α,12α-triHCA	NA	No
7α,12α-Dihydroxy-3-oxocholest-4-en-26-oic acid*^d^*	7α,12α-diH,3O-CA	LMST04030150	No
Trihydroxy-3-oxocholest-4-en-26-oic acid		NA	No
7α-Hydroxy-3,24-bisoxocholest-4-en-26-oic acid*^d^*	7αH,3,24-diO-CA	NA	No
7α-Hydroxy-26-nor-cholest-4-ene-3,24-dione*^d^*	7αH,26-nor-C-3,24-diO	NA	No
3β,7α-Dihydroxychol-5-en-24-oic acid	3β,7α-Δ^5^-BA	LMST04010217	Yes
7α-Hydroxy-3-oxochol-4-en-24-oic acid	7αH,3O-Δ^4^-BA	LMST04010239	Yes
3β,7β-Dihydroxychol-5-en-24-oic acid	3β,7β-Δ^5^-BA	LMST04010218	Yes

NA, not applicable.

a Location of double bonds unknown.

b The 7-DHC isomerizes to 8-DHC.

c Dehydration product of cholestane-3β,5α,6β-triol.

d Authentic standard not available; annotation based on retention time, exact mass, and MS^3^ fragmentation.

e The 3β,7α-diHCA dehydrates to 3βH-7-DHCA.

f The 7αH,3O-CA dehydrates to 3O-6-DHCA.

g Sum of intact 3β,7α-diHCA and its dehydration product 3βH-7-DHCA.

h Sum of intact 7αH,3O-CA and its dehydration product 3O-6-DHCA.

i Identification based on comparison to 7α-isomer.

## MATERIALS AND METHODS

### Human samples

Serum was from 35 patients diagnosed with ALS (24 male, 11 female, mean age 65), 6 patients diagnosed with primary lateral sclerosis (PLS; 2 male, 4 female, mean age 69), and 24 control samples (12 male, 12 female, mean age 58). CSF was from ALS patients (n = 20; 15 male, 5 female, mean age 61) and controls (n = 15; 12 male, 3 female, mean age 75). Serum and CSF samples were obtained from ALS and PLS patients and healthy volunteers (typically patient spouses and friends) as part of The Oxford Study for Biomarkers in Motor Neuron Disease (BioMOx). Two expert neurologists (M.R.T. and K.T.) made the diagnosis of ALS and PLS according to standard criteria. All participants provided informed consent, and the study was approved by the South Central Oxford Ethics Committee B. Additional control CSF samples for method development were from a study performed at Methodist Hospital, Houston, TX.

### Methods

#### Serum.

One hundred microliters of serum was added drop-wise to a solution of 1,050 µl of absolute ethanol containing 2 ng of 25-[26,26,26,27,27,27-^2^H_6_]hydroxyvitamin D_3_ (25-[^2^H_6_]D_3_, Toronto Research Chemicals, Ontario, Canada), 2 ng of 7α,25-[26,26,26,27,27,27-^2^H_6_]dihydroxycholesterol (7α,25-[^2^H_6_]diHC), 20 ng of 7α-[25,26,26,26,27,27,27-^2^H_7_]hydroxycholesterol (7α-[^2^H_7_]HC), 20 ng of 24R/S-[25,26,26,26,27,27,27-^2^H_7_]hydroxycholesterol (24R/S-[^2^H_7_]HC), 20 ng of 22R-[25,26,26,26,27,27,27-^2^H_7_]hydroxycholset-4-en-3-one (22R-[^2^H_7_]HCO), and 20 µg of [25,26,26,26,27,27,27-^2^H_7_]cholesterol (deuterated sterols from Avanti Polar Lipids Inc., Alabaster, AL) with sonication in an ultrasonic bath. After 5 min, 350 µl of water was added to make the solution 70% ethanol. After a further 5 min of sonication, the solution was centrifuged at 17,000 *g* at 4°C for 30 min.

To separate cholesterol from the more polar oxysterols and steroid acids, the sample solution, now in 1.5 ml of 70% ethanol, was loaded onto a 200-mg Certified Sep-Pak C_18_ column (Waters, Elstree, Herts, U.K.) preconditioned with 4 ml of absolute ethanol and 6 ml of 70% ethanol. The solvent flow rate through the column was at a rate of ∼0.25 ml/min assisted by negative pressure at the column outlet generated by a vacuum manifold. The flow-through (1.5 ml) was combined with a column wash of 70% ethanol (5.5 ml) to give fraction SPE1-Fr1 (7 ml) in which 25-D_3_, oxysterols, cholestenoic, and cholenoic acids elute. A second fraction (SPE1-Fr2) was collected by eluting with a further 4 ml of 70% ethanol, before fraction 3 containing cholesterol and sterols of similar hydrophobicity was eluted with 2 ml of absolute ethanol (SPE1-Fr3). Finally, a fourth fraction eluting in 2 ml of absolute ethanol was collected, containing lipid more hydrophobic than cholesterol (SPE1-Fr4). Each fraction was divided into two equal subfractions, (A) and (B), and allowed to dry overnight under vacuum. Note that a hydrolysis step was not included in the sample preparation procedure, and by removing cholesterol from its metabolites in the first step of sample preparation, subsequent confusion with ex vivo oxidation products is eliminated.

Lyophilized material was reconstituted in 100 µl of propanol-2-ol. Each sample was vortexed thoroughly. To subfractions (A) 1,000 µl of 50 mM phosphate buffer (pH 7) was added, containing 3.0 µl of cholesterol oxidase from *Streptomyces* sp. (2 mg/ml in H_2_O, 44 units/mg protein, Sigma-Aldrich Ltd, Dorset, England). The reaction mixture was left at 37°C for 1 h, after which the reaction was quenched with 2,000 µl of methanol. Subfractions (B) were treated identically to subfractions (A), but in the absence of cholesterol oxidase. One hundred fifty microliters of glacial acetic acid was added to subfractions (A) and (B) and vortexed. One hundred ninety milligrams of [^2^H_5_]Girard P (GP) reagent, bromide salt (23), was added to subfractions (A), 150 mg of [^2^H_0_]GP reagent, chloride salt (TCI Europe, Zwijndrecht, Belgium), was added to subfractions (B) to allow for subsequent simultaneous LC/MS analysis; see below (supplemental Fig. S1). After vortexing, the derivatization reaction was left to proceed at room temperature, overnight in the dark.

To remove excess derivatization reagent, a 60-mg Oasis HLB (Waters) column was conditioned with 6 ml of 100% methanol, 6 ml of 10% methanol, and 4 ml of 70% methanol before the samples (3,250 µl, ∼70% organic) were loaded. The sample vessel was washed with 1 ml of 70% methanol, and the wash was applied to the column. One milliliter of 35% methanol was used to rinse the column, and the combined 5 ml of effluent was diluted with 4 ml of water to give 9 ml of 35% methanol solution. This solution was reapplied to the column, followed by a rinse of the column with 1 ml of 17.5% methanol. The 10 ml of combined effluent was diluted with 9 ml of water to give a 19-ml solution of 17.5% methanol. Reapplying the 19-ml solution to the column completed the recycling procedure by which sterols, oxysterols, cholestenoic, and cholenoic acids were extracted by the column, whereas derivatization reagents eluted to waste. Six milliliters of 10% methanol was added to wash the column. Finally, 25-D_3_, oxysterols, cholestenoic and cholenoic acids, and more nonpolar sterols were eluted from the column with 3 × 1 ml 100% methanol (SPE2-Fr1,2,3) and 1 ml of absolute ethanol (SPE2-Fr4). Oxysterols, 25-D_3_, cholestenoic, and cholenoic acids elute in the first 2 ml from the column (SPE2-Fr1,2). Cholesterol and sterols of similar polarity elute over 3 ml of methanol (SPE2-Fr1,2,3).

For 25-D_3_, oxysterol, cholestenoic, and cholenoic acid analysis, just before analysis by LC/MS, an equal aliquot of SPE2-Fr1,2 from subfractions (A) and (B) was combined together in one tube. For cholesterol analysis equal volumes of SPE2-Fr1,2,3 from subfractions (A) and (B) were combined. In both cases the combined effluent in methanol was diluted with water to achieve a concentration of 60% methanol, i.e., the initial composition of the mobile phases during LC analyses.

#### LC/MS and LC/MS^n^ analysis.

Analysis was performed on a LTQ-Orbitrap Elite (Thermo Fisher Scientific, Hemel Hempstead, UK) equipped with an electrospray probe, and a Dionex Ultimate 3000 LC system (Dionex; now Thermo Fisher Scientific), essentially as described previously (22, 23). For each injection, three to five scan events were performed: one high-resolution (120,000, full-width at half maximum height at *m/z* 400) MS scan event in the Orbitrap analyzer in parallel with two to four multistage fragmentation (MS^n^) scan events in the LTQ linear ion trap. Quantification was performed by stable isotope dilution or by using isotope-labeled structural analogs.

#### CSF.

Two hundred fifty microliters of CSF was added drop-wise to 2,100 µl of absolute ethanol containing 2 ng of 24R/S-[^2^H_7_]HC, 2 ng of 7α-[^2^H_7_]HC, 2 ng of 22R-[^2^H_7_]HCO, 0.4 ng of 7α,25-[^2^H_6_]diHC, 4 ng of 25-[^2^H_6_]D_3_, and 0.8 µg of [^2^H_7_]cholesterol with sonication in an ultrasonic bath. Six hundred fifty microliters of water was added, and the solution was sonicated for 5 min, followed by centrifugation at 2,400 *g* at 4°C for 30 min.

The CSF sample solution in 3 ml of 70% ethanol was loaded onto a 200-mg Certified Sep-Pak C_18_ column as in the serum protocol. However, the 3-ml flow-through was combined with a column wash of only 4 ml of 70% ethanol to give SPE1-Fr1 (7 ml) in which 25-D_3_, oxysterols, cholestenoic, and cholenoic acids elute. The remainder of the sample preparation procedure, including derivatization, was identical to that used for serum, with the exception that the final eluates from SPE2 were lyophilized and reconstituted in 60% methanol prior to sample injection on the LC system.

### Statistics

An ANOVA was run for each sterol. Univariate *t* tests were performed against the control group: *P < 0.05; **P < 0.01. Concentrations given in the text are means ± SDs.

## RESULTS

### Analysis of sterols, oxysterols, and cholestenoic and cholenoic acids in serum

Using enzyme-assisted derivatization for sterol analysis technology (22) in combination with LC/MS with MS^3^, we analyzed serum from 35 patients diagnosed with ALS (24 male, 11 female, mean age 65) and 24 control samples (12 male, 12 female, mean age 58). Six patients diagnosed with the upper motor neuron-only, very slowly progressive variant of ALS, termed PLS (2 male, 4 female, mean age 69), were separately compared with the control group. Concentrations of nonesterified cholesterol, two of its precursors, and >40 metabolites were measured (see Table 1 for compounds and abbreviations and supplemental Table S1 for quantitative data; note that a hydrolysis step was not carried out, so all concentrations are for nonesterified compounds). We failed to find any sexual dimorphism in the control sample set and thus included both sexes in the current study.

**Fig. 1.**
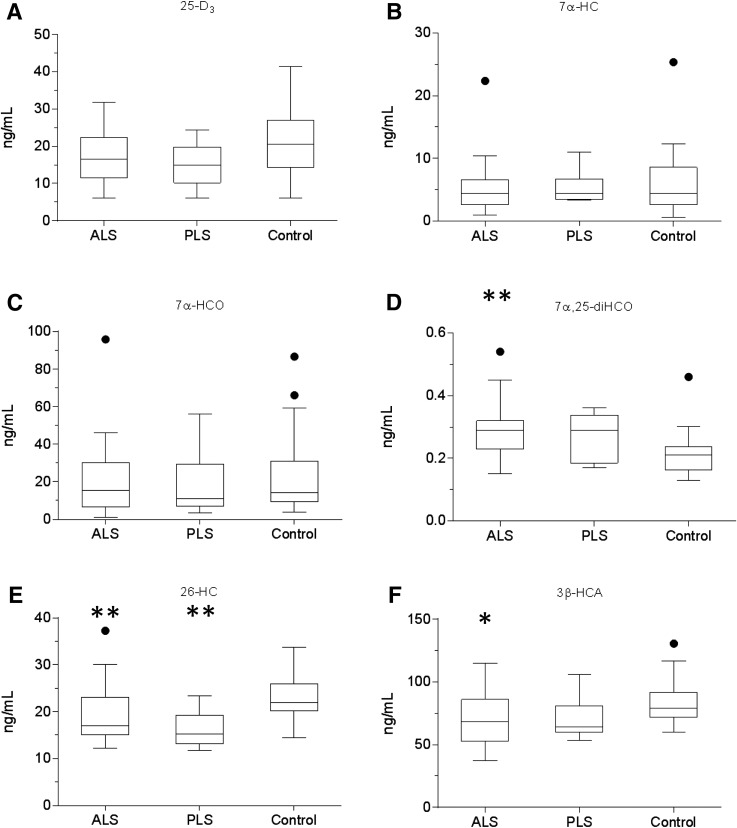
Concentration of 25-D_3_ and cholesterol metabolites in serum. Box and whiskers plots showing the concentrations (ng/ml) of 25-D_3_ (A), 7α-HC (B), 7α-HCO (C), 7α,25-diHCO (D), 26-HC (E), and 3β-HCA (F) in serum from ALS (n = 35) and PLS (n = 6) patients and healthy controls (n = 24). The bottom and top of the box are the first and third quartiles, and the band inside the box represents the median. The whiskers extend to the most extreme data points, which are no more than 1.5 times the range between first and third quartile distant from the box. Points beyond that are plotted individually. Data for other sterols can be found in supplemental Table S1. Univariate *t* tests were performed against the control group. * *P* < 0.05; ** *P* < 0.01.

There was no significant difference in the concentrations of cholesterol or its precursors desmosterol (24-DHC) or 7-dehydrocholesterol (7-DHC) between ALS, PLS, or control samples. Note we report here the concentration of 7-DHC as the sum of 7-DHC and its isomer 8-DHC, which are only partially resolved on our LC system. The 8-DHC is an enzymatic product of 7-DHC (24). Similarly, there was no significant difference in the concentration of the 7-DHC metabolite 25-hydroxyvitamin D_3_ (25-D_3_) in serum of the ALS patients compared with controls (**Fig. 1**).

**Fig. 2.**
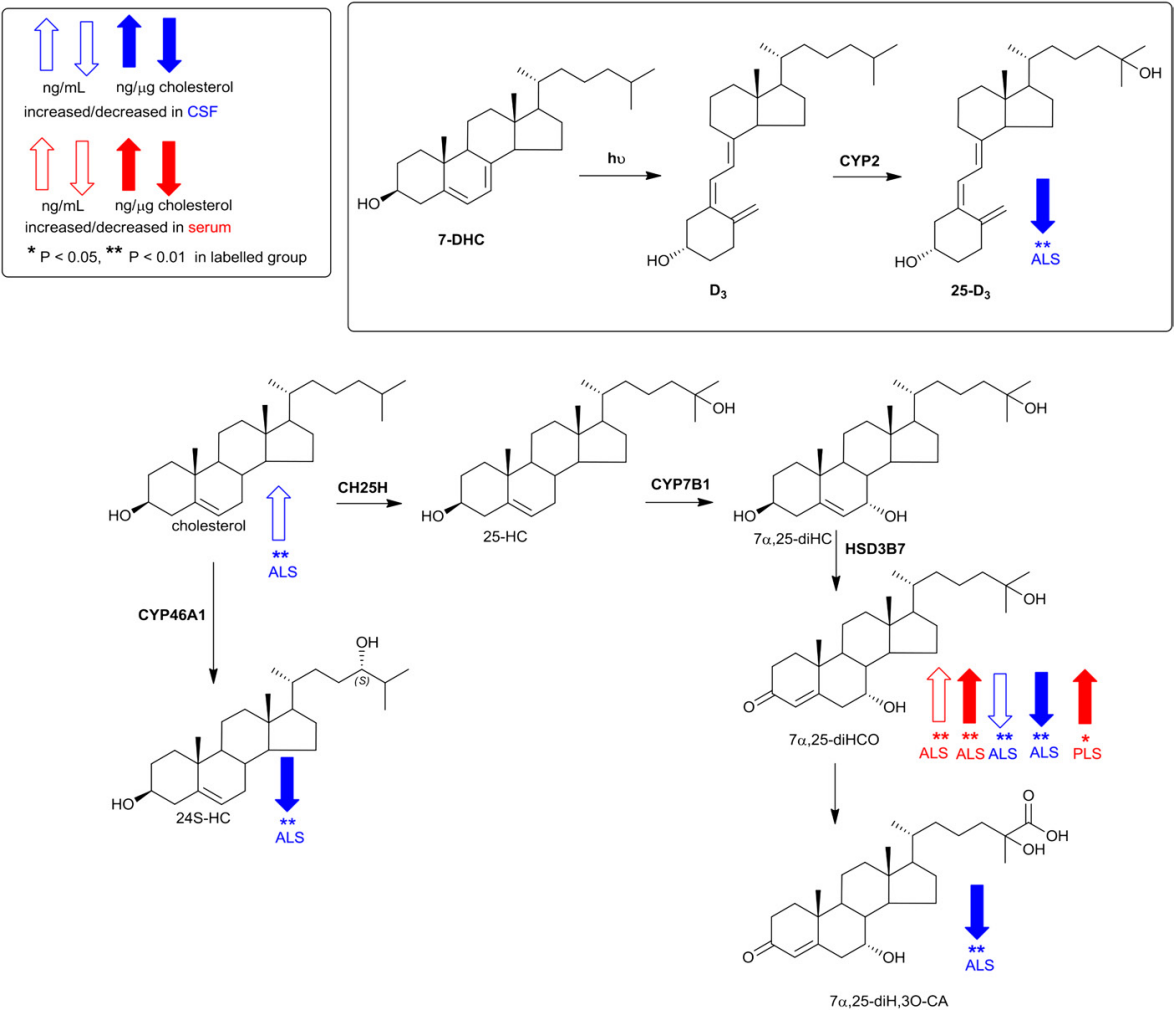
Pathways of cholesterol metabolism initiated by the enzymes cholesterol 25-hydroxylase (CH25H) and CYP46A1. Changes in sterols concentration in CSF and serum are indicated by blue and red arrows, respectively. The direction of change corresponds to the direction of the arrow. Enzymes catalyzing the indicated reactions are shown where known. Univariate *t* tests were performed against the control group. * *P* < 0.05; ** *P* < 0.01.

**Fig. 3.**
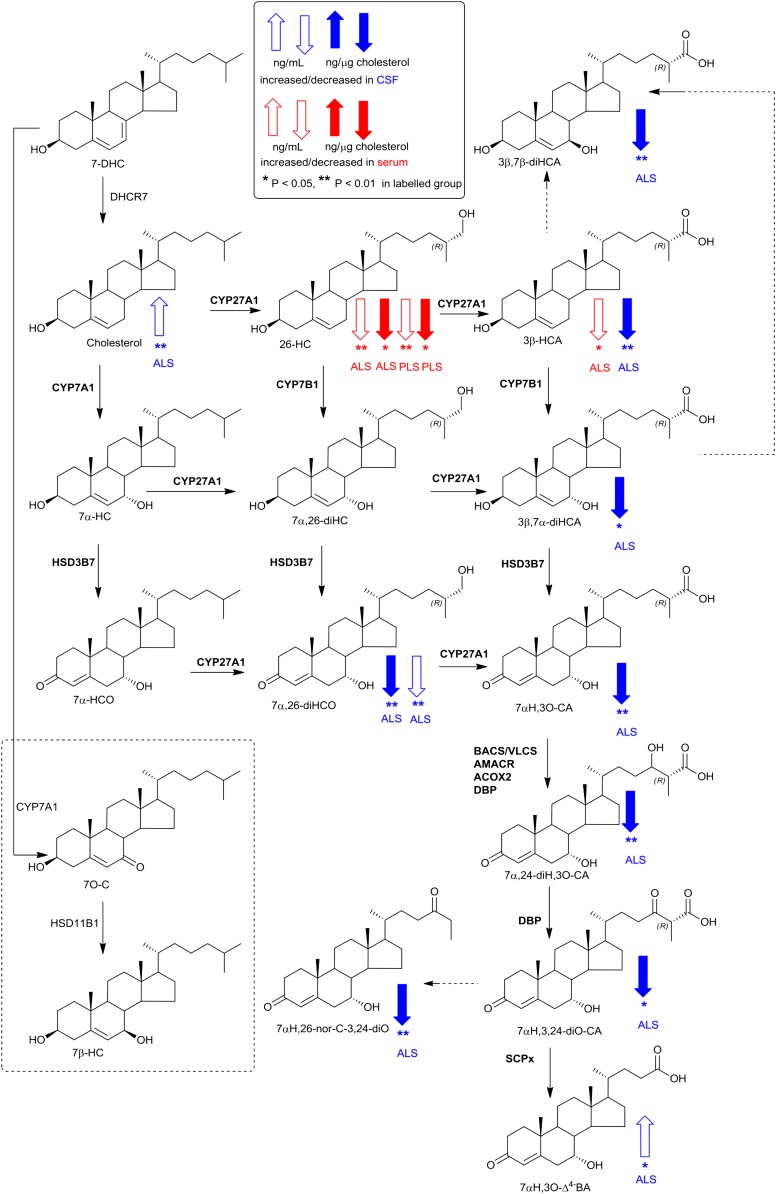
Pathway of cholesterol metabolism initiated by the enzymes CYP7A1 and CYP27A1. Changes in sterols concentration in CSF and serum are indicated by blue and red arrows, respectively. The direction of change corresponds to the direction of the arrow. Enzymes catalyzing the indicated reactions are shown where known. Enzyme abbreviations used are as follows: ACOX2, acyl-CoA oxidase 2, branched chain; AMACR, α-methylacyl-CoA racemase; BACS, bile acyl-CoA synthetase; DBP, D-bifunctional protein or multifunctional enzyme type 2 (HSD17B4); SCPx, sterol carrier protein x; VLCS, very long chain acyl-CoA synthetase. Univariate *t* tests were performed against the control group. * *P* < 0.05; ** *P* < 0.01.

The first steps of all cholesterol metabolism lead to hydroxycholesterol isomers, collectively known as oxysterols. A minor pathway, initiated in activated macrophages, leads to 25-hydroxycholesterol (25-HC), followed by subsequent metabolism to 7α,25-dihydroxycholesterol (7α,25-diHC) and 7α,25-dihydroxycholest-4-en-3-one (7α,25-diHCO, **Fig. 2**). Of these metabolites, 7α,25-diHCO was elevated in ALS serum (*P* < 0.01). 7α-Hydroxycholesterol (7α-HC) and 7α-hydroxycholest-4-en-3-one (7α-HCO) are the first members of the neutral pathway of bile acid biosynthesis (**Fig. 3**) (25); however, neither oxysterol showed a difference in concentration between ALS, PLS, or control samples, and nor did 7β-hydroxycholesterol (7β-HC) or 7-oxocholesterol (7O-C). 7O-C, 7α-HC, and 7β-HC can be formed via free radical oxidation of cholesterol, and these metabolites have been suggested as markers of oxidant stress (26).

Conversely, (25R)26-hydroxycholesterol (26-HC), the first member of the extrahepatic part of the acidic pathway of bile acid biosynthesis, was decreased in ALS and PLS serum compared with controls (*P* < 0.01), as was 3β-hydroxycholest-5-en-26-oic acid (3β-HCA) in ALS serum (*P* < 0.05). CYP27A1 is the enzyme responsible for hydroxylation of cholesterol at (25R)C-26 and then subsequent oxidation to the (25R)26 carboxylic acid. Note that we use the systematic nomenclature (27), where hydroxylation of the terminal carbon of the cholesterol side chain introducing 25R stereochemistry results in (25R)26-hydroxycholesterol (28). Unless stated otherwise, R stereochemistry is generally assumed at C-25. The nonsystematic name for 26-HC, widely used in the literature on account of the nomenclature of its synthesizing enzyme, CYP27A1, is 27-hydroxycholesterol. Levels of 26-HC in the circulation are often correlated to cholesterol, with high cholesterol levels associated with high levels of 26-HC (29). We thus decided to normalize for each sample the level of 26-HC and other metabolites to cholesterol to determine whether the observed differences were still maintained. When normalized to cholesterol (supplemental Table S2), elevation in concentration of 7α,25-diHCO was retained in ALS, as was a decrease in the concentration of 26-HC. Interestingly, in an earlier study, Wuolikainen et al. (30) found that “total” 26-HC in male ALS patients was similarly reduced. In combination, these results suggest reduced transcription, translation, or activity of cholesterol (25R)26-hydroxylase in ALS.

### Analysis of sterols, oxysterols, and cholestenoic and cholenoic acids in CSF

**Fig. 4.**
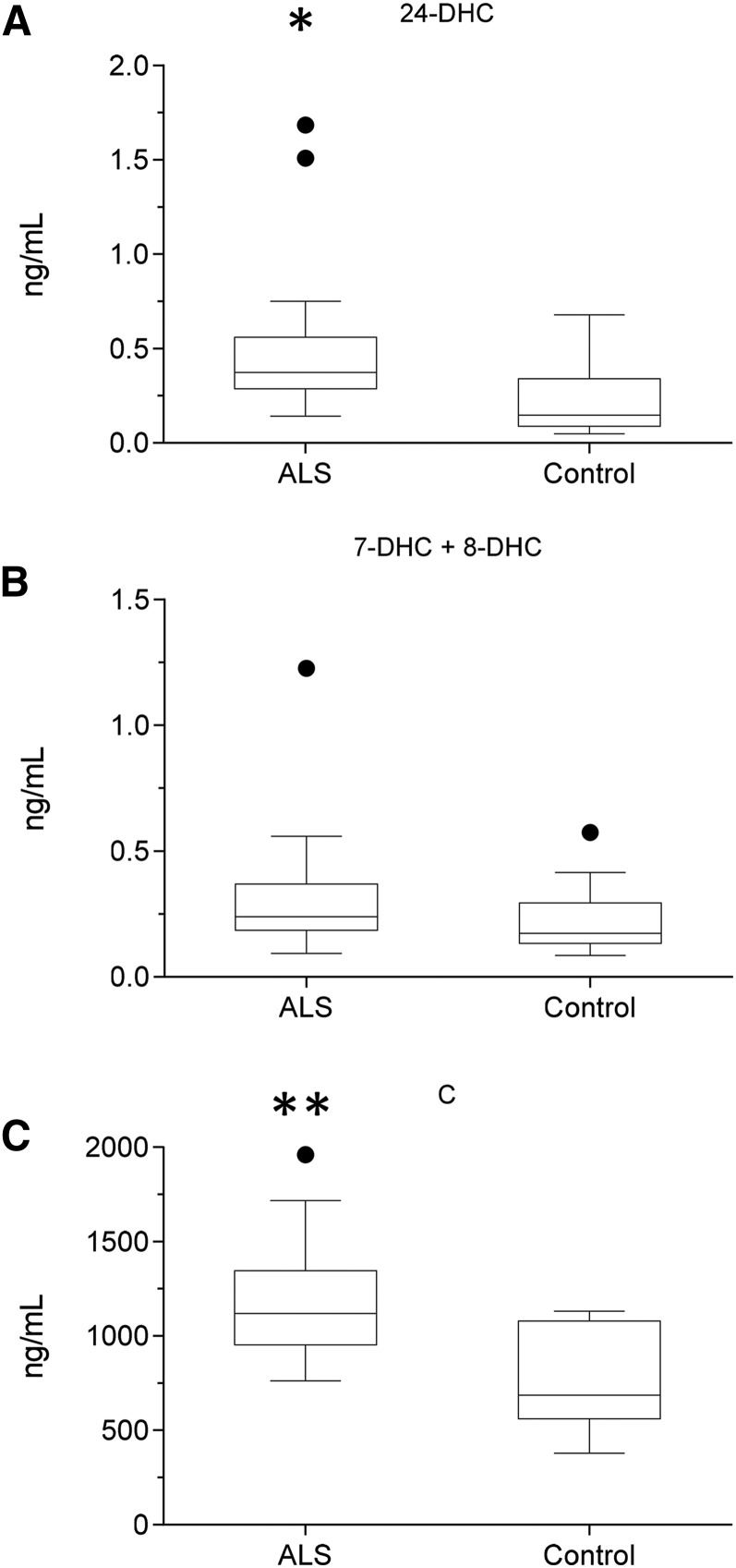
Concentration of cholesterol and its precursors in CSF. Box and whisker plots showing concentrations (ng/ml) of 24-DHC (A), 7-DHC + 8-DHC (B), and cholesterol (C) in CSF from ALS (n = 20) patients and healthy controls (n = 15). Box and whiskers are as described in the Fig. 1 legend. Data for other sterols can be found in supplemental Table S3. Univariate *t* tests were performed against the control group. * *P* < 0.05; ** *P* < 0.01.

**Fig. 5.**
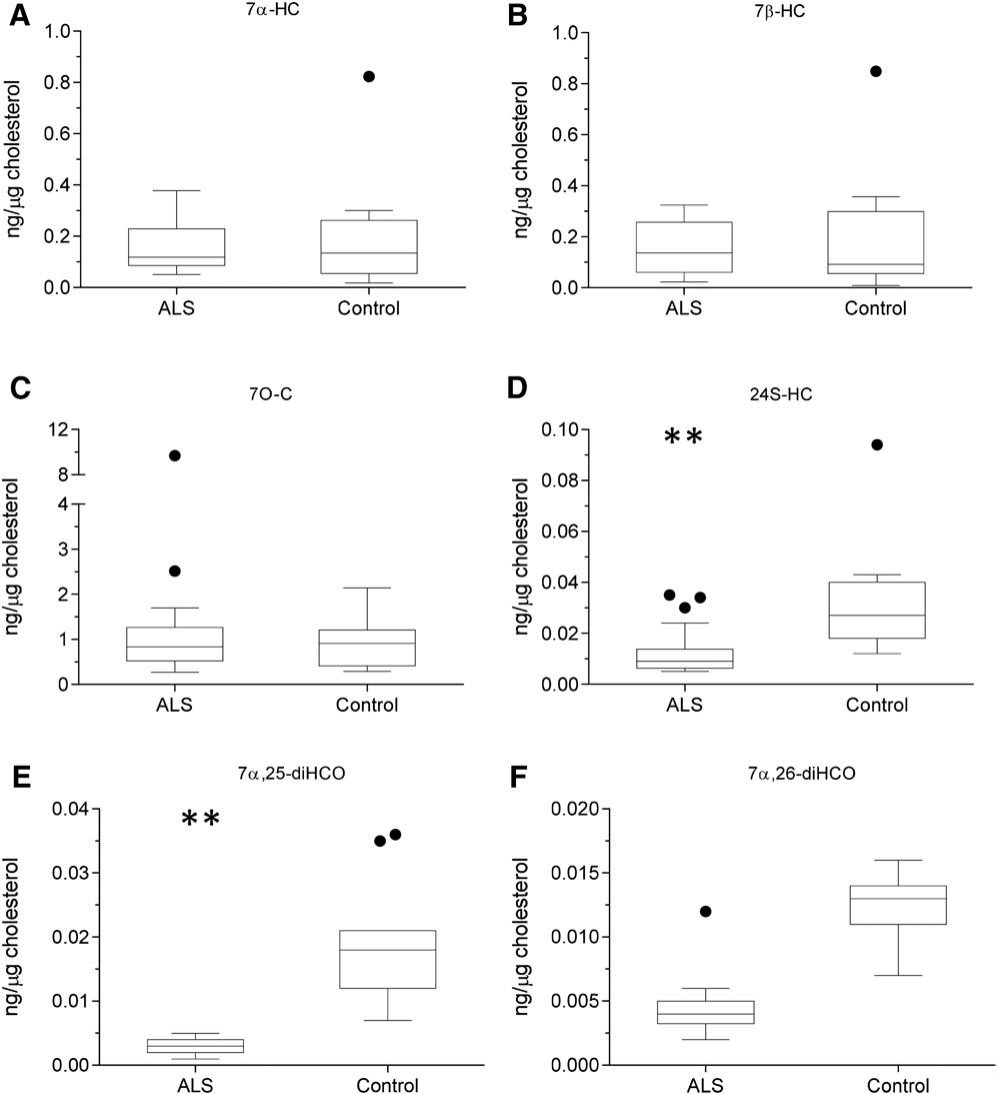
Concentration of CYP7A1, CYP46A1, and CH25H pathway metabolites in CSF. Box and whisker plots showing concentrations (ng/µg cholesterol) of 7α-HC (A), 7β-HC (B), 7O-C (C), 24S-HC (D), 7α,25-diHCO (E), and 7α,26-diHCO (F) in CSF from ALS (n = 20) patients and healthy controls (n = 15). Box and whiskers are as described in the Fig. 1 legend. Data for other sterols can be found in supplemental Table S4. Univariate *t* tests were performed against the control group. * *P* < 0.05; ** *P* < 0.01.

**Fig. 6.**
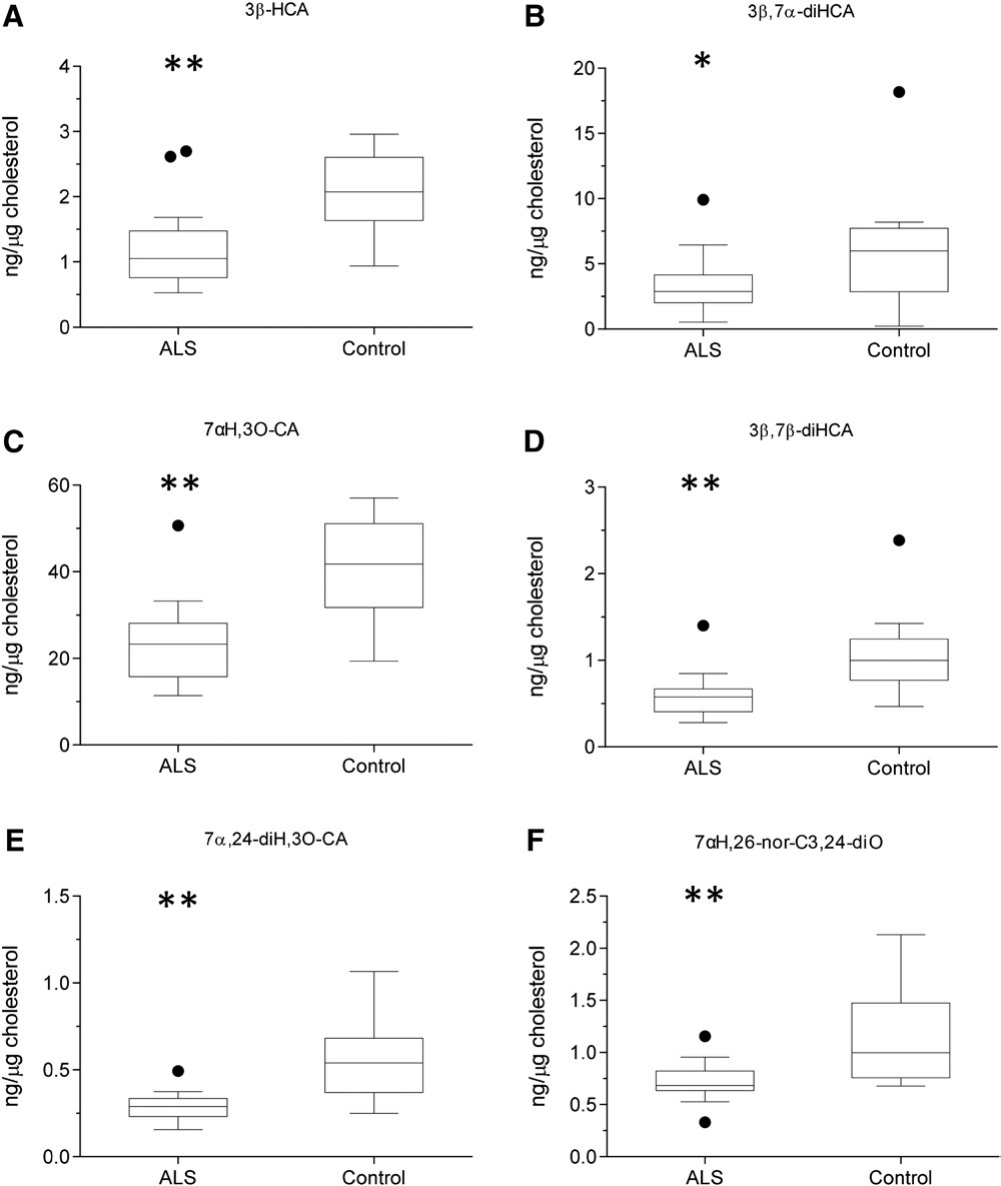
Concentration of acidic pathway metabolites in CSF. Box and whisker plots showing concentrations (ng/µg cholesterol) of 3β-HCA (A), 3β,7α-diHCA (B), 7αH,3O-CA (C), 3β,7β-diHCA (D), 7α,24-diH,3O-CA (E), and 7αH,26-nor-C-3,24-diO (F) in CSF from ALS (n = 20) patients and healthy controls (n = 15). Box and whiskers are as described in the Fig. 1 legend. Data for other sterols can be found in supplemental Table S4. Univariate *t* tests were performed against the control group. * *P* < 0.05; ** *P* < 0.01.

As with serum, levels of cholesterol, 7-DHC (plus 8-DHC), and desmosterol were measured in CSF from ALS patients (n = 20; 15 male, 5 female, mean age 61) and controls (n = 15; 12 male, 3 female, mean age 75). The concentrations of both desmosterol (*P* < 0.05) and cholesterol (*P* < 0.01) were found to be elevated in CSF from ALS patients (**Fig. 4**, supplemental Table S3). Because ALS is a neurodegenerative disease, we speculated that, when cholesterol is released by neurons as they die, it would be metabolized by CYP46A1 to 24S-hydroxycholesterol (24S-HC) and by CYP27A1 to members of the acidic pathway of bile acid biosynthesis (Figs. 2 and , respectively) (25). Surprisingly, when normalized to cholesterol, 24S-HC concentration was found to be reduced (*P* < 0.01) in CSF, as were members of the acidic pathway of bile acid biosynthesis (supplemental Table S4). Intriguingly, concentrations of 7α-HC, 7α-HCO, 7β-HC, and 7O-C, which originate from the CYP7A1-initiated arm of the bile acid biosynthesis pathway and enter the CSF from the circulation, did not differ between ALS patients and controls (**Fig. 5**). This can be explained because CYP7A1 is not expressed in the CNS, being liver-specific (Fig. 3) (25). The level of 26-HC was not significantly lower in CSF from ALS patients (0.09 ± 0.06 ng/µg cholesterol) compared with controls (0.13 ± 0.07 ng/µg cholesterol), but its downstream metabolites 3β-HCA (*P* < 0.01), 3β,7α-dihydroxycholest-5-en-26-oic acid (3β,7α-diHCA; *P* < 0.05), and 7α-hydroxy-3-oxocholest-4-en-26-oic acid (7αH,3O-CA; *P* < 0.01) were all reduced in concentration (ng/µg cholesterol) in ALS CSF (**Fig. 6**). Whereas (25R)26-carboxylation by CYP27A1 occurs in the mitochondria, 7α-hydroxylation by CYP7B1, the oxysterol 7α-hydroxylase, and oxidation of the 3β-hydroxy group by hydroxysteroid dehydrogenase (HSD) 3B7 occur in the endoplasmic reticulum, further metabolism to bile acids proceeds in the peroxisome (31). Peroxisomal metabolites found in CSF include 7α,24-dihydroxy-3-oxocholest-4-en-26-oic acid (7α,24-diH,3O-CA); 7α-hydroxy-3,24-*bis*oxocholest-4-en-26-oic acid (7αH,3,24-diO-CA), which is also observed as the decarboxylated dione; 7α-hydroxy-26-*nor*cholest-4-ene-3,24-dione (7αH,26-nor-C-3,24-diO); and the β-oxidation product 7α-hydroxy-3-oxochol-4-en-24-oic acid (7αH,3O-Δ^4^-BA). With the exception of the latter compound, all of the peroxisomal intermediates were decreased (*P* < 0.05 or 0.01; ng/µl cholesterol) in CSF from ALS patients.

## DISCUSSION

The major route for cholesterol metabolism in the CNS is conversion to 24S-HC via the neuron-specific enzyme CYP46A1 (32, 33), accounting for approximately two-thirds of cholesterol metabolism in brain, and elevated levels of total 24S-HC have been found in CSF of patients with some neurodegenerative diseases, e.g., Alzheimer’s disease (AD) (34, 35). The term total describes the sum of both esterified and nonesterified sterol. In most studies, concentrations of total cholesterol or of total hydroxycholesterols are measured where sterols esterified with fatty acids are hydrolyzed and the total sterol is assayed, although it is the nonesterified sterols that are biologically active. Usually the ratio of esterified to nonesterified sterol in plasma is approximately 10:1 (36). Surprisingly, levels of total 24S-HC are decreased in blood plasma of patients with advanced AD, presumably as a consequence of a reduced number of active neurons producing 24S-HC (37). In a recent study, Wuolikainen et al. found total 24S-HC plasma levels to be increased in female ALS patients, but not in male patients, whereas total 26-HC was reduced in male ALS patients, but not females (30). Side-chain hydroxycholesterols, i.e., 22R-HC, 24S-HC, 25-HC, and 26-HC, and also cholestenoic acids, are ligands to liver X receptors (LXRs), the β-isoform being particularly expressed in brain, and, interestingly, LXRβ^−/−^ mice show adult-onset motor neuron pathology (38, 39). Importantly, LXRβ is involved in control of lipogenesis and cholesterol homeostasis, and these mice showed increased cholesterol levels in spinal cord, gliosis, and inflammation preceding motor neuron loss and clinical disease onset (40). In the present study, a loss of LXR signaling may result from reduced CNS levels of oxysterol and cholestenoic acid LXR-ligands, ultimately leading to motor neuron pathology.

Although cholesterol cannot cross the blood-brain barrier and be imported to, or exported from, the brain, 24S-HC, 26-HC, and other cholesterol metabolites can cross the blood-brain barrier (35, 41). Heverin et al. (42) have shown that 26-HC is imported to the brain, whereas Meaney et al. (43) have shown that its acidic metabolite 7αH,3O-CA is exported from the brain to circulation. Further studies by Iuliano et al. (44) have shown that a precursor of this acid, 7α,26-dihydroxycholest-4-en-3-one (7α,26-diHCO), is also exported from the brain to circulation, as is its isomer 7α,25-diHCO. In another study, Ogundare et al. (45) have shown that multiple parts of the acidic pathway of bile acid biosynthesis are active in the CNS.

**Fig. 7.**
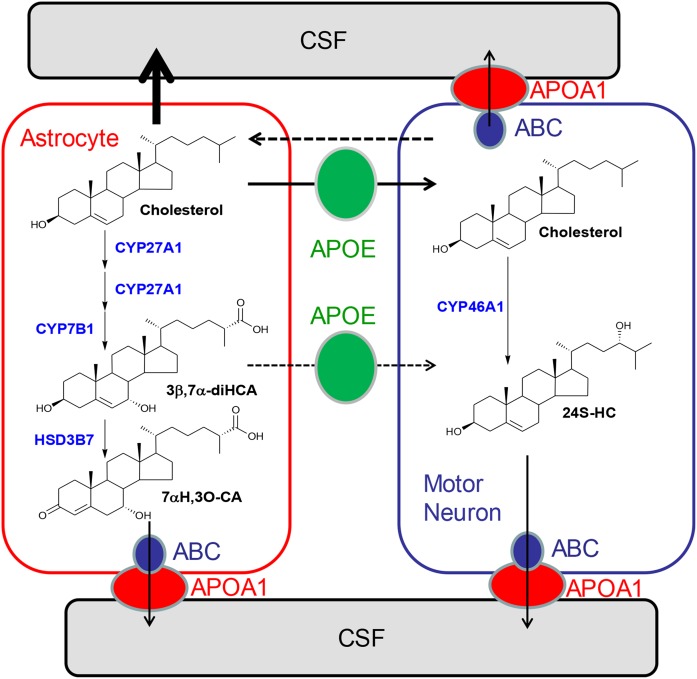
Hypothetical model of cholesterol homeostasis in neurons and astrocytes. Neurons import cholesterol from astrocytes mediated by apolipoprotein (APO) E (green circle) (53). Neurons dispose of cholesterol by ATP-binding cassette (ABC) transporters (blue circle) and APOA1 (red circle), by the formation of 24S-HC, or via return to astrocytes via an unknown mechanism (broken arrow). Activation of LXRβ by oxysterols or cholestenoic acids leads to increased expression of ABC transporters and increased sterol release. Impaired metabolism of cholesterol in astrocytes as suggested in the present study of ALS patients may result in a greater flux of cholesterol out of astrocytes into the CSF and also into neurons. A reduction of metabolism of cholesterol to cholestenoic acids will result in a decrease in LXRβ ligands in astrocytes and lower amounts transported to neurons (broken arrow depicts unknown mechanism). A consequence will be increased cellular levels of cholesterol in neurons and reduced antiinflammatory signaling by LXRβ.

A key finding of the present study was the increase in concentration of nonesterified cholesterol in the CSF of ALS patients (Fig. 4). This increase was associated with a decrease in the cholesterol-normalized concentration of brain-derived oxysterols and cholestenoic acids, but not of those cholesterol metabolites imported from the circulation (Figs. 5 and ). One explanation for increased cholesterol levels in CSF of ALS patients is that cholesterol is released from membranes as neurons die, and metabolic pathways are saturated and insufficient to remove this excess in ALS patients (**Fig. 7**). An alternative explanation is that there is dysregulated transport of sterols between neurons and glia, resulting in inefficient cholesterol metabolism in the CNS of ALS patients as a consequence of ineffective LXR signaling. This latter hypothesis is supported by studies on the LXRβ^−/−^ mouse, which not only shows adult motor neuron pathology, but also increased spinal cord cholesterol levels, accumulation of cholesterol in ventral horn neurons, gliosis, and inflammation preceding motor neuron loss and clinical disease onset (40). How LXRβ protects against motor neuron loss is not exactly clear, but LXRβ is known to attenuate the inflammatory response (46), and neuroinflammation is an important pathogenic factor in ALS (47). In fact, proinflammatory cytokines and monocyte chemotactic protein 1 were increased in LXRβ^−/−^ mice, suggesting that LXRβ plays a fundamental role in the CNS by reducing levels of cytokines and therefore development of neuroinflammation (40).

Importantly, the oxysterol 24S-HC and the cholestenoic acids 3β-HCA, 3β,7α-diHCA, and 3β,7β-diHCA, each found to be reduced in CSF from ALS patients, are LXR ligands (48). Thus, neuroinflammation in ALS may be associated with a reduction in CNS LXR ligands and, consequently, LXR signaling. In fact, 3β,7α-diHCA has been ascribed a neuroprotective role toward developing motor neurons through the LXRs, although the other two acids appear to be neurotoxic (48). One explanation for reduced levels of LXR ligands in the CSF of ALS patients is that, as neurons die in ALS, less of the neuron-specific endoplasmic reticulum enzyme CYP46A1 is available to metabolize cholesterol to 24S-HC, thereby accounting for its reduced concentration in the CSF. Other LXR ligands found in the CSF, including 3β-HCA, 3β,7α-diHCA, and 3β,7β-diHCA, are derived from cholesterol in astrocytes (49). The first enzyme in their synthesis from cholesterol is mitochondrial CYP27A1 (Fig. 3). Interestingly, *CYP27A1* has been identified as a susceptibility gene for sporadic ALS (13). Mutations in *CYP27A1* leading to a defective CYP27A1 enzyme are the cause of the genetic neurodegenerative disorder cerebrotendinous xanthomatosis (50).

Further support for a role for oxysterols in neuroinflammation comes from the recent report by Reboldi et al. linking side-chain hydroxylated cholesterol molecules (i.e., 25-HC) to inhibition of *Il1b* transcription and inflammasome activity by antagonizing the sterol regulatory element-binding protein-driven cholesterol biosynthesis pathway (51). Importantly, it has been shown that 24S-HC will also inhibit SREBP-driven cholesterol biosynthesis (52), so, conversely, reduced levels of 24S-HC in the CNS, as observed in ALS, should result in enhanced cholesterol biosynthesis and *Il1b* transcription, inflammasome activity, and neuroinflammation.

In summary, we have identified elevated cholesterol concentrations in the CSF of ALS patients. When normalized to cholesterol, concentrations of glial-derived acidic metabolites are reduced, but cholesterol metabolites imported from the circulation are not. These data point to impaired activity of the CYP27A1 enzyme leading to a failure of the CNS to remove excess cholesterol, which may be toxic to neuronal cells, compounded by a reduction in neuroprotective 3β,7α-diHCA and other LXR ligands (Fig. 7).

## Supplementary Material

Supplemental Data
