# Supplementary material for: Defective cholesterol metabolism in amyotrophic lateral sclerosis
Source: J Lipid Res. 2016 Dec 29;58(1):267–78. doi: 10.1194/jlr.P071639 (PMC5234729; doi:10.1194/jlr.P071639)
Supplement: Supplemental Data [file supp_58_1_267__index.html]

Defective cholesterol metabolism in amyotrophic lateral sclerosis — Defective cholesterol metabolism in amyotrophic lateral sclerosis — Supplemental Data 

# Defective cholesterol metabolism in amyotrophic lateral sclerosis

## Supplemental Data

- Supplemental Figure S1 (.pdf, 119 KB) - Charge-tagging approach to sterol analysis by LC-MS
- Supplemental tables S1-S4 (.xlsx, 88 KB) - Concentrations of sterols, oxysterols, cholestenoic and cholenoic acids in serum and CSF
