# Supplementary figures and images for: Defective cholesterol metabolism in amyotrophic lateral sclerosis
Source: J Lipid Res. 2016 Dec 29;58(1):267–78. doi: 10.1194/jlr.P071639 (PMC5234729; doi:10.1194/jlr.P071639)

# Supplemental Figure S1 (Griffiths)

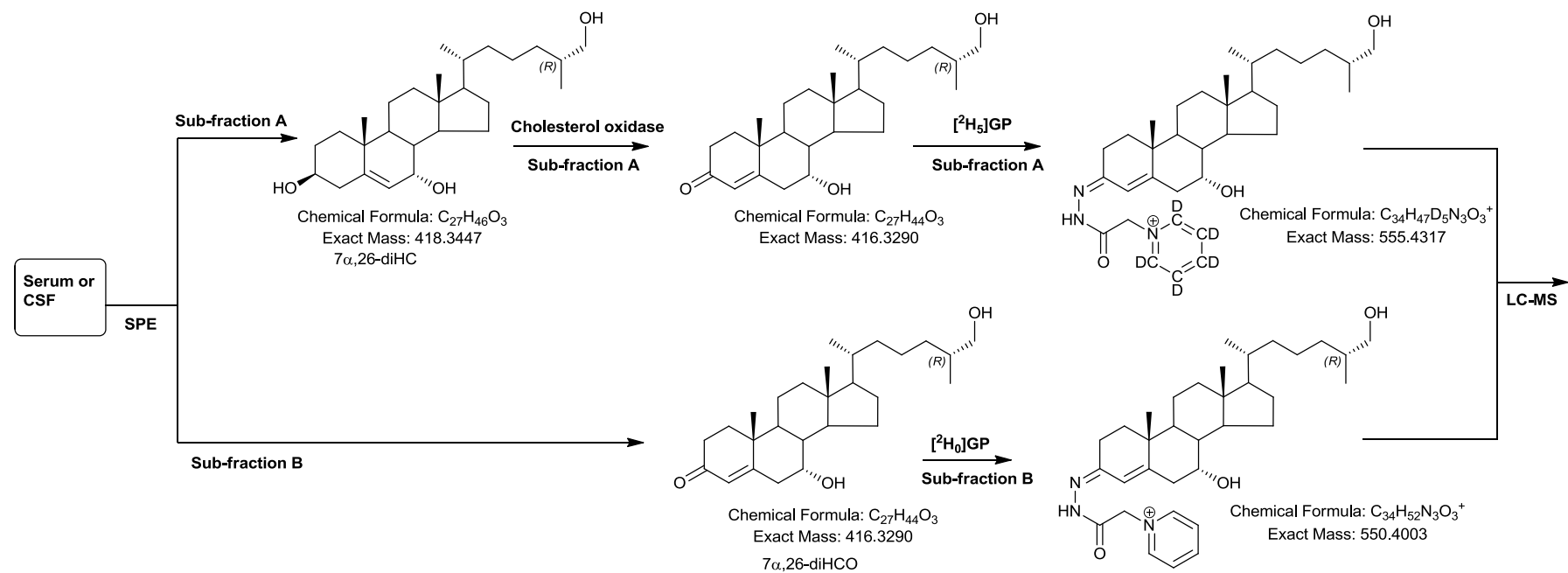

Supplement: Supplemental Data [file 10.1194_P071639_jlr.P071639-1.pdf]
